# Optimising Beam Geometry in Orthopaedic X‐Rays: A Phantom Study

**DOI:** 10.1002/jmrs.895

**Published:** 2025-06-03

**Authors:** Jodie Ringin, Zac Maibaum, Lucy Fox, Will Merritt

**Affiliations:** ^1^ Deakin University Australia

## Abstract

**Introduction:**

Antoni Cieszynski, a 19th‐century Polish dentist, pioneered a rule of isometry for accurate dental radiography. His bisecting angle technique improved the precision of imaging teeth by angling the x‐ray tube perpendicular to a ‘bisecting line’ between the object and the image receptor, thereby minimising geometric distortion and achieving more anatomically accurate images. This study explored the potential use of this rule for x‐ray imaging of long bones when routine positioning techniques are compromised.

**Methods:**

Using an adult phantom forearm, an experiment was conducted to quantify the effectiveness of the bisecting angle technique on a long bone. A control image using a straight tube with the bone parallel to the digital image receptor (IR) was taken. This was followed by x‐rays of the phantom forearm at 15° increments with a straight tube, matching tube angle and the bisecting angle technique. The resulting images were analysed by a single scribe and reviewed by one other peer for geometric distortion using the calliper measurement tool.

**Results:**

The control image measured 21.4 cm. When the central ray matched the angle of the phantom forearm, the maximum elongation exceeded the length of the detector (> 43 cm). When a straight tube angle was applied, the maximum foreshortening measured 13.5 cm. Meanwhile, the maximum length of the phantom forearm, when the bisecting angle was applied, was 24.1 cm. While the use of the bisecting angle technique did not eliminate geometric distortion, it greatly reduced it. The experiment identified that common radiographic practice has the potential to be improved.

**Conclusions:**

The bisecting angle technique offers a promising method to improve long bone imaging. While it does not fully eliminate geometric distortion, it effectively minimises elongation, suggesting a potential to enhance imaging accuracy for long bones in clinical settings.

## Graphical abstract

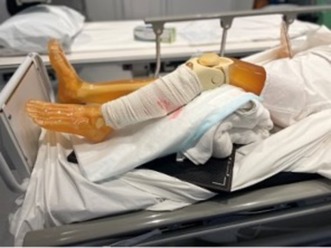

Antoni Cieszynski, a 19th‐century Polish dentist, developed the bisecting angle technique in 1907, which uses isometry rules to improve anatomical imaging accuracy in dentistry. Our experiment tested this technique on the radius bone at various angles, demonstrating that it significantly reduced elongation and distortion compared to traditional methods, though not entirely eliminating them. These findings suggest that the bisecting angle could offer a further recognised imaging technique in radiography for the imaging of long bones.

## 1 Introduction

While technology continues to revolutionise the diagnostic capabilities of medical imaging, the challenge for a radiographer to position a patient who presents with challenging clinical conditions remains. Diagnostic radiography is underpinned by the fundamental principles of geometry to obtain anatomically accurate images [1]. One of the challenges a radiographer faces is imaging patients who, due to their injuries or condition, cannot be positioned in a traditional manner [1]. This necessitates innovative approaches to adaptive positioning techniques to ensure diagnostic quality, while accommodating patient limitations.

Antoni Cieszynski, a distinguished Polish academic and dentist born in 1882, made notable contributions to imaging through his pioneering work in dental x‐ray [2]. Cieszyński's published work, particularly his research on radiographic positioning techniques and the development of isometric principles in dental imaging, highlights his contribution to improving the accuracy and diagnostic quality of dental radiographs [3].

In the early 20th century, Cieszynski developed a technique to address inherent geometric distortion in dental radiography caused by the limited space in the oral cavity. His solution, the bisecting angle technique, involves angling the x‐ray tube perpendicular to a ‘bisecting line’ between the object and the image receptor, thereby minimising geometric distortion and achieving more anatomically accurate images [3, 4]. This principle, while primarily applied in dental imaging, holds potential benefits for broader radiographic practices [5].

**FIGURE 1:**
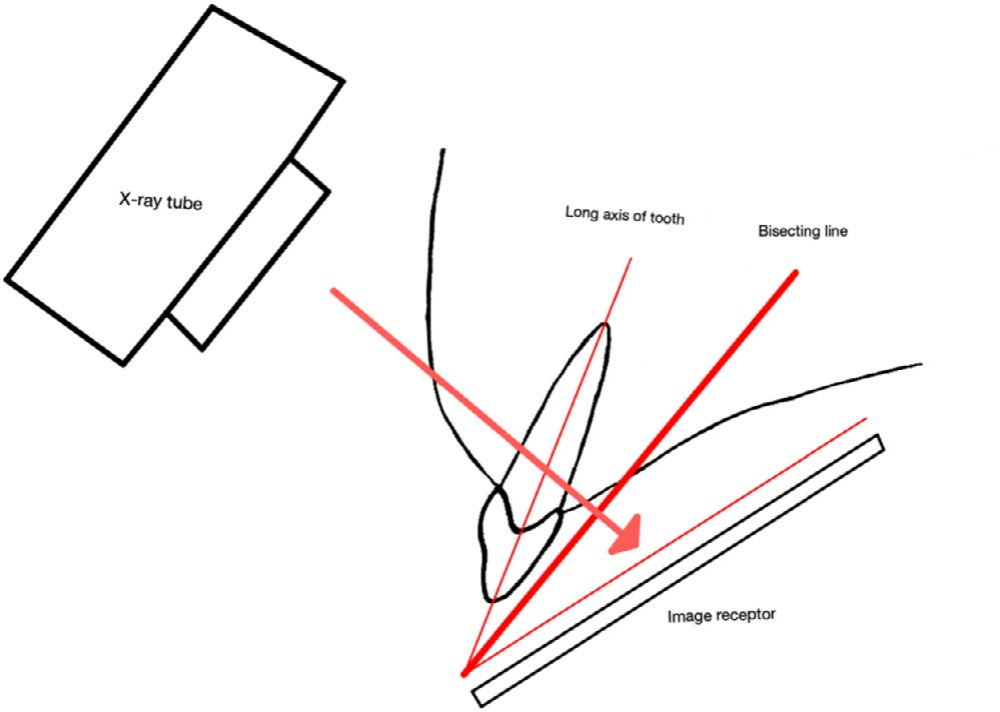
Depiction of the bisecting angle technique used in dental radiography (adapted from Dental Radiography, University of Edinburgh) [6].

Cieszynski's technique, originally designed for dental imaging, leverages geometric principles to achieve accurate anatomical representations despite challenging positioning, see Figure 1. The technique's foundation is simple, yet profound: by splitting a triangle in half, with a central bisecting line and the central ray angled perpendicular to this line, the resultant image ensures minimal distortion to the long bone [4].

**FIGURE 2:**
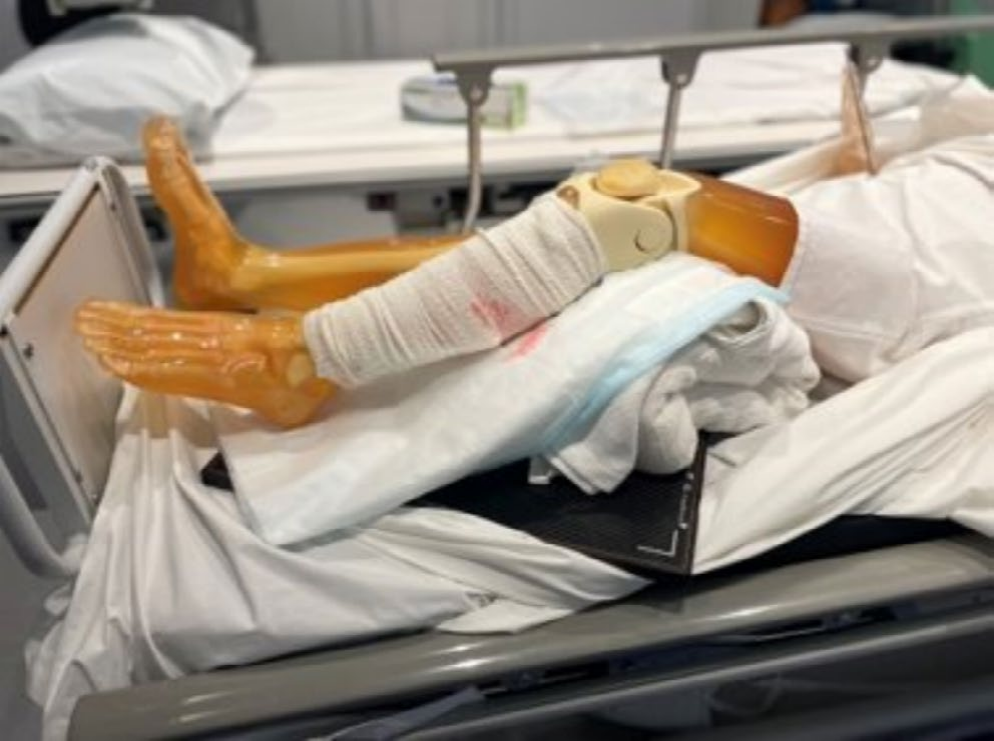
Simulated trauma presentation.

In clinical radiography, ensuring the anatomy is parallel to the image receptor can be unfeasible. For example, a patient with severe fractures and pain in the lower leg—like the phantom patient seen in Figure 2—may not be able to straighten their leg for a standard AP tib/fib projection [1]. Traditional imaging in such scenarios can result in geometric distortion, either through foreshortening or elongation of the imaged bone.

This challenge inspired an investigation into the application of the bisecting angle technique to modern day radiography. Through a phantom forearm study, the bisecting angle was explored to quantify if it could be adapted to image long bones accurately when traditional positioning is not achievable due to the patient's clinical condition.

The hypothesis of the study was that the bisecting angle technique can produce reduced geometric shape distortion compared to that of conventional techniques.

**FIGURE 3:**
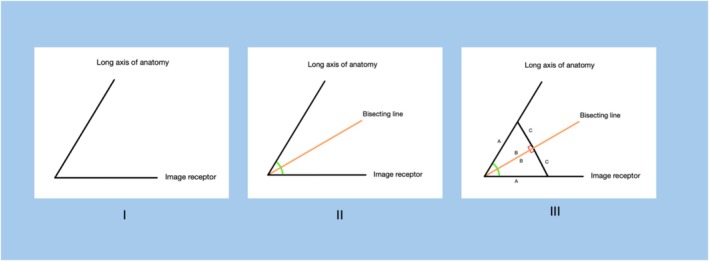
Pictorial explanation for Cieszynski's rule of isometry.

Figure 3 provides a clear pictorial explanation of how Cieszynski's isometry technique works. The diagram in picture **i** depicts the geometric relationship between the image receptor (IR) and a long bone. Image **ii** shows how two congruent angles can be formed. The two angles marked are identical and split by a centre line. This is called the bisecting line. By adding perpendicular lines across the bisecting line, depicted in image **iii**, two identical right‐angle triangles are created. Basic laws of geometry state that a triangle's angles sum to 180°.

With the bisecting line, the two triangles that are created are symmetrical and will have identical angles. This means the hypotenuse (A) of each triangle, representing the anatomy and IR, is of equal length and elongation/foreshortening is eliminated.

## 2 Materials and Methods

A phantom study was conducted using a Carestream Ascend floor mounted digital radiography (DR) x‐ray system [7]. The phantom forearm was from a Somso skeleton [8], which had been disconnected from the humerus for ease of placement and repetition. Ethics approval was not required.

**FIGURE 4:**
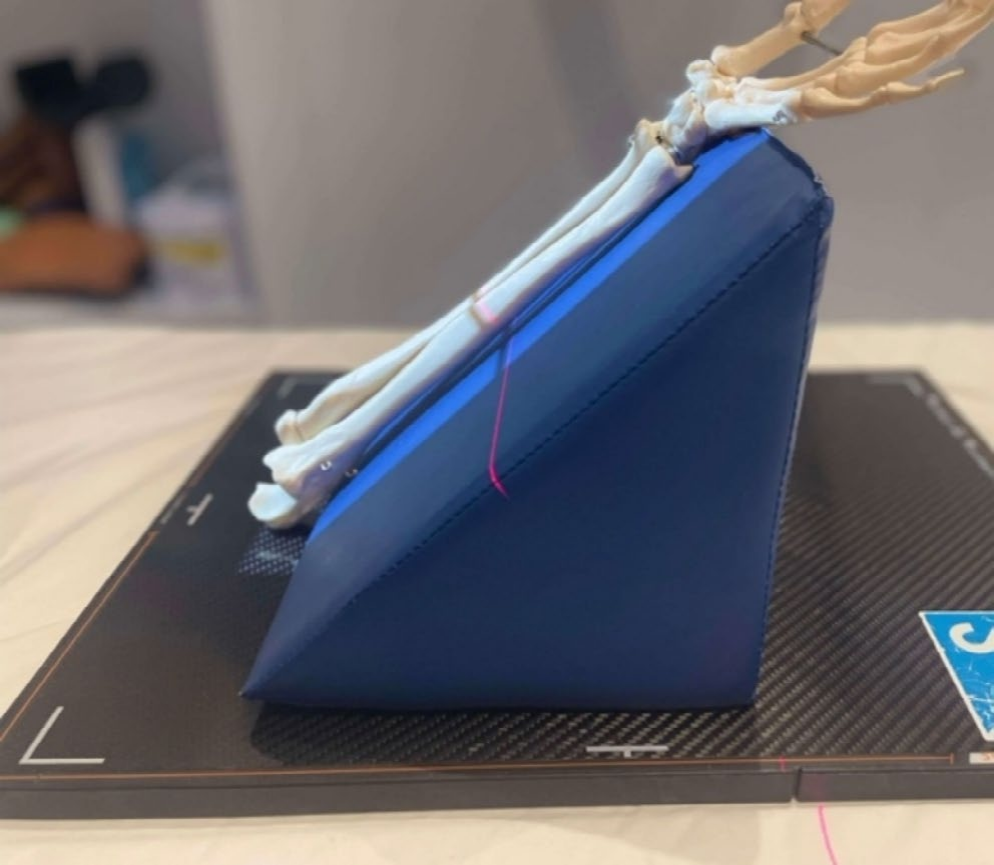
A clinical sponge is used to demonstrate the angulation of the phantom forearm.

All 13 images were taken under the following conditions: 100 cm source‐to‐image distance (SID), 1.6 mA‐second (mAs) and a kilovoltage peak (kVp) of 60. The same phantom forearm was used throughout the experiment with clinical radiographic sponges of angles at 15° increments from 15°–60° inclusive, see Figure 4.

To begin, a control image was obtained with the phantom forearm placed parallel to the image receptor with a 0° tube angle perpendicular to the IR. To measure the length of the bone, the calliper post‐processing tool on the console was used. The measurement was taken from the most prominent part of the radial tuberosity to the tip of the radial styloid process. This length was 21.4 cm. All succeeding images were measured from this point because it is the most pronounced and is easily identifiable as the angle of the phantom forearm increases.

Following the control image, x‐rays of the forearm at 15° increments were taken. Clinical sponges were used to achieve angling of the forearm at 15°, 30°, 45° and 60°. For each angle, three images were taken. The first image utilised a straight tube, the second image matched the tube angle with the angle of the anatomy and finally, the third image used the bisecting angle technique. For example, the bisecting angle technique meant that if the forearm was on a 30° sponge, a 15° tube angle was used. For each image, the length of the radius was recorded by a single scribe and reviewed by one other peer.

With the data, a percentage distortion relative to the true radius length (21.4 cm) was calculated. This was calculated by subtracting the true length from the measured length, dividing the result by the true length and then multiplying by 100 to express the result as a percentage. This formula determines the deviation between the measured and true lengths, expressing it as a percentage of the true length for data analysis.

## 3 Results

The results, as shown in Table 1, demonstrate observable variation in the length of the radius depending on the tube angle technique applied. The use of a straight tube resulted in foreshortening at angles greater than 15° and matching the tube angle to the anatomy caused elongation. The true length of the radius measured 21.4 cm. Maximum measured distortion occurred at 60°, with the radius measuring 13.5 cm with a straight tube and a length that exceeded the IR when a matching tube angle was applied. The bisecting angle technique consistently produced less distortion, more closely approximating the true length of the radius. The greatest difference occurred at 60°, with the radius length measuring 24.1 cm, 2.7 cm longer than the actual length. The general trend of the dataset demonstrates that matching the tube angle to the angle of the anatomy incurs elongation, while utilising a straight tube resulted in foreshortening, but the use of the bisecting angle technique yields minimal distortion.

**TABLE 1:** Results of the radius length measurement with the forearm phantom angled at 0°, 15°, 30°, 45° and 60° to the image receptor (IR).

Angle of bone (degrees)	Length with bisecting tube angle (cm)	Length with straight tube (cm)	Length when matching angle of bone (current practice) (cm)
**0**	—	21.4	—
**15**	22.3	21.6	23.7
**30**	23.7	20.9	28.2
**45**	23.6	17.5	36.3
**60**	24.1	13.5	> 43 (Detector Size)

**FIGURE 5:**
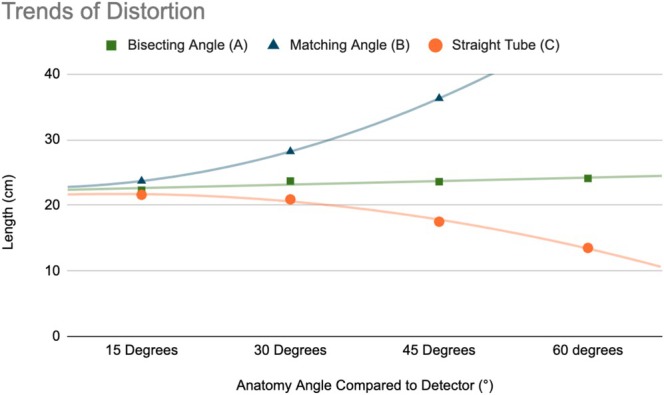
Line graph shows trends of distortion as the angle of anatomy compared to the image receptor (IR) increases, when the bisecting angle (A), matching tube angle (B) and straight tube angle (C) are applied.

The trends of the dataset are illustrated in Figure 5. Using a straight tube results in progressive foreshortening, while matching the tube angle to the anatomy leads to elongation, with distortion increasing exponentially as the angle deviates from parallel.

**FIGURE 6:**
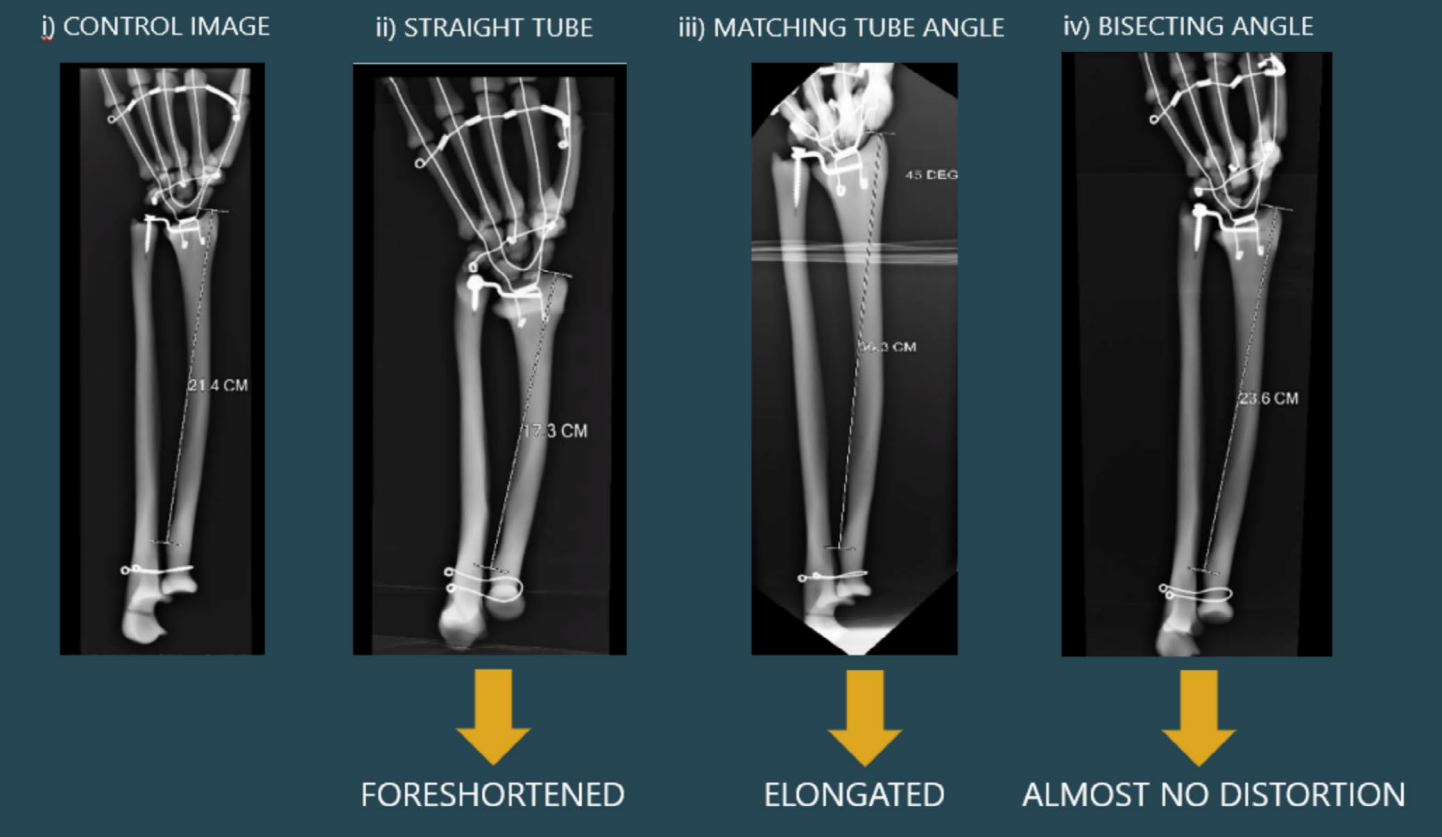
(i) Shows the control image with anatomy parallel to image receptor (IR) and straight tube angle, compared to the radius at a 45° angle with a straight tube angle (ii), matching tube angle (iii) and the bisecting angle technique (iv).

Figure 6 ii demonstrates the technique's effectiveness, where the phantom forearm is 45° from the image receptor. When a straight tube is used, the bone on the radiograph measures 17.3 cm, equal to 5 cm of foreshortening compared to its actual length. On the contrary, if the angle of the tube is matched to the angle of the bone—a technique which is commonly practiced—it becomes 36.3 cm long on the image, due to distortion. While if the bisecting angle technique is implemented, when the phantom forearm is 45° from the IR and the x‐ray tube is angled 22.5°, the bone measures 23.6 cm, just 2 cm longer than its actual length.

## 4 Discussion

The results of this study demonstrate a clear relationship between radiographic technique and the degree of geometric distortion when measuring bone length at increasing angles. The percentage distortion analysis reveals that each method introduces characteristic measurement errors, each with implications for accuracy in clinical radiography.

The bisecting angle technique consistently overestimated bone length, with geometric distortion ranging from +4.2% at 15° to +12.6% at 60°. While this method does not eliminate distortion, the relative consistency of overestimation suggests that it provides a more stable approach across various positions. These findings align with the theoretical principles of the bisecting angle method, which aims to balance elongation and foreshortening by directing the central ray perpendicular to the bisecting plane between the bone and the IR.

Maintaining a straight tube caused foreshortening for angles of 30° and greater, which increased exponentially as the angle increased, measuring −2.3% at 30°, −18.2% at 45° and − 36.9% at 60°. At 15°, however, an outlier occurs: the measured radial length is slightly elongated, deviating from the overall trend of foreshortening. This may be due to the combined effects of object‐to‐image distance (OID) and the divergence of the x‐ray beam. While trigonometric alignment based solely on the central ray would suggest foreshortening, the diverging beam after it intersects with the forearm may introduce slight magnification, resulting in this image appearing elongated compared to the control (0°). It appears there may be a threshold between 15° and 30° where this magnification effect temporarily outweighs the foreshortening of the anatomy. Despite this outlier, the overall dataset continues to demonstrate that using a straight tube when the anatomy is not parallel to the image receptor introduces progressive foreshortening. This finding highlights the limitations of the straight tube approach in imaging obliquely positioned structures.

The current clinical practice of matching the tube angle to the bone angle produced the greatest measurement errors, particularly at higher forearm phantom angles. While geometric distortion remained moderate at 15° (+10.7%), it increased sharply at 30° (+31.8%) and 45° (+69.6%), exceeding the detector size limit at 60°. The extreme elongation observed is attributed to the alignment of the x‐ray beam with the long axis of the bone, maximising magnification effects. These findings suggest that it is unsuitable for accurate length assessment due to excessive geometric distortion.

Overall, the bisecting angle technique demonstrated the most stable distortion pattern across increasing forearm phantom angles, while the straight tube and matching tube angle methods exhibited increasing underestimation and overestimation, respectively. These findings support reconsidering the bisecting angle approach as a tool for when the standard imaging approach is not attainable. Future research should explore how these geometric distortion patterns influence clinical decision‐making and whether refinements to the bisecting angle method could further optimise radiographic accuracy.

### 4.1 Clinical Application

Radiographers encounter numerous clinical scenarios where a patient cannot be positioned with their anatomy parallel to the image receptor. This might occur not only in trauma cases but also in situations involving patients experiencing extreme pain, deformities, post‐surgical positioning restrictions or other conditions. These challenges can lead to patient discomfort and often result in a suboptimal image with geometric distortion. For angles of 15° or greater, the bisecting angle technique offers a more reliable alternative by reducing foreshortening while keeping overestimation within a predictable range. This may be important when accurate length assessment is required, such as in pre‐surgical planning.

It is acknowledged that image distortion encompasses multiple factors, including magnification, shape distortion (elongation/foreshortening) and blurring (penumbra, motion artefacts and edge unsharpness). This study specifically focused on the effect of beam geometry on shape distortion in our chosen phantom forearm image, particularly elongation and foreshortening, rather than the broad range of image quality parameters.

### 4.2 Limitations

While the experiment provided notable data, the results are not without limitations. As many aspects as possible were controlled throughout the experiment, such as the SID and exposure factors. The x‐ray machinery receives regular servicing from an authorised provider. However, there is no additional quality assurance testing outside of this, which could be noted as a limitation for this study. To acquire the relevant angles, 15° and 45° angled clinical sponges were used. It is noted that these sponges are not a recognised measuring tool, though is commonly used equipment in a modern hospital setting.

Measurements were kept as consistent as possible using the most prominent point of the radial tuberosity as the proximal landmark and the tip of the radial styloid process as the distal landmark, though this is still a subjective measurement that needs to be accounted for when reviewing the results. The experiment is limited by the fact that it involved just one anatomical region; it was not repeated and involved a small sample size. Despite these limitations, the results demonstrate a clear trend in the data and the potential for the bisecting angle technique to be implemented into clinical practice upon further and more stringent scientific testing with large sample sizes.

## 5 Conclusion

This study highlights the effectiveness of Cieszynski's bisecting angle technique in preventing geometric distortion in the radiography of long bones, providing radiographers with an additional tool.

Further research into the bisecting angle technique could change long bone radiography in the future, ensuring that radiographers can provide the best possible diagnostic images, with precision and minimal discomfort to their patients, even when met with challenging circumstances.

## Conflicts of Interest

The authors declare no conflicts of interest.

## Data Availability

The data that support the findings of this study are available from the corresponding author upon reasonable request.
